# Efficacy of Transarterial Chemoembolization Combined with Tyrosine Kinase Inhibitors for Hepatocellular Carcinoma Patients with Portal Vein Tumor Thrombus: A Systematic Review and Meta-Analysis

**DOI:** 10.3390/curroncol30010096

**Published:** 2023-01-16

**Authors:** Jia Deng, Ziyue Liao, Jian Gao

**Affiliations:** 1 Second Clinical College, Chongqing Medical University, Chongqing 400000, China; 2 Department of Gastroenterology, The Second Affiliated Hospital of Chongqing Medical University, Chongqing 400000, China

**Keywords:** hepatocellular neoplasm (HCC), portal vein tumor thrombus (PVTT), transcatheter chemoembolization (TACE), tyrosine kinase inhibitor (TKI), meta-analysis

## Abstract

Background: Transarterial chemoembolization (TACE) combined with tyrosine kinase inhibitors (TKIs) may enhance the efficacy of treatment for hepatocellular carcinoma (HCC) with portal vein tumor thrombus (PVTT); however, it remains unclear. We aim to evaluate the efficacy of TACE combined with TKIs. Methods: A thorough literature search was performed on major databases since their inception until October 2022. Based on the eligibility criteria, eight studies (2103 patients) were included. Results: Meta-analysis showed that TACE+sorafenib/apatinib had a better tumor response (objective response rate (ORR): RR = 4.85, 95% CI 2.68–8.75, disease control rate (DCR): RR = 3.23, 95% CI 1.88–5.56), and prolonged OS (HR = 0.50, 95%CI 0.42–0.60, *p* < 0.00001) than TACE alone. TACE+lenvatinib was stronger than TACE+sorafenib in ORR (60.7% vs. 38.9%) and TTP (HR = 0.61, 95% CI 0.43–0.86), whereas it was similar in DCR (96.4% vs. 96.3%) and OS (HR = 0.70 95% CI 0.46–1.05). Conclusions: TACE plus sorafenib or apatinib was superior to TACE alone for hepatocellular carcinoma with PVTT; no significant advantage was found between TACE+lenvatinib and TACE+sorafenib, although TACE+lenvatinib performed better in terms of ORR and TTP.

## 1. Introduction

Hepatocellular carcinoma (HCC) is one of the most malignant tumors and the third leading cause of cancer-related deaths worldwide [1]. Portal vein tumor thrombus (PVTT) is considered a feature of advanced HCC and is usually associated with a very poor prognosis [2]. Surgical treatments such as liver transplantation and hepatectomy also have limited efficacy [3].

For patients with unresectable HCC, transcatheter chemoembolization (TACE) is the most common treatment according to the Barcelona Clinic Liver Cancer (BCLC) treatment strategy [4], and some studies have suggested that patients with intact liver function and portal vein collateral circulation at thrombosis would benefit from TACE [5,6]. However, the hypoxic environment caused by TACE also promotes the expression of vascular endothelial growth factor (VEGF), which may lead to tumor angiogenesis and local recurrence [7,8,9].

Tyrosine kinase inhibitors (TKIs) inhibit tumor vascular growth through multiple pathways, including inhibition of the VEGF receptor. Sorafenib, the first oral TKI approved for advanced HCC, prevents tumor growth by inhibiting the intracellular signaling pathways and extracellular receptors, such as the VEGF receptor and platelet-derived growth factor (PDGF) receptors [10,11]. However, the efficacy of sorafenib combined with TACE remains controversial owing to drug resistance and safety issues in recent years [12,13]. The emergence of novel tyrosine kinase inhibitors has provided new options. Lenvatinib, another first-line agent, targets receptor tyrosine kinase (RTK), such as VEGFR, fibroblast growth factor receptors (FGFR), and PDGFR [14,15]; while apatinib particularly binds to VEGFR-2 [16]. In addition, cabozantinib, regorafenib, and donafenib have been adopted for the treatment of HCC in the last few years [17]. Therefore, it can be assumed that TACE plus tyrosine kinase inhibitors (TKIs) could decrease tumor microvessel density by inhibiting the hypoxia-induced overexpression of VEGF. Combination therapy may enhance the efficacy of patients with HCC and PVTT.

To date, several studies have indicated that the combination of immune checkpoint inhibitors (ICIs) with TACE has shown acceptable survival benefit [18,19], while severaltrials on combinational therapy of TACE plus monoclonal antibodies to VEGF have exposed the short-term efficacy [20,21]. Phase II clinical trials evaluating the safety and efficacy of TACE in combination with anti-PD-L1 antibody atezolizumab and the anti-VEGF bevacizumab are ongoing [22,23]. In addition, trials about the targeted delivery of adequate glucose oxidase (GOX) into tumor cells by covalent organic frameworks (COFs) have been successful in mice [24,25,26].

Nevertheless, the therapeutic measures for HCC that accompany PVTT are debatable, and the effectiveness and safety of tyrosine kinase inhibitors plus TACE remain unclear. 

Therefore, our purpose was to assess the efficacy of TKI agents in combination with TACE and the relative efficacy of novel TKI agents plus TACE to provide some bias in the treatment of patients with HCC and PVTT.

## 2. Materials and Methods

This meta-analysis study was conducted and reported in accordance with the Preferred Reporting Items for Systematic Reviews and Meta-Analysis (PRISMA) 2020 guidelines [27]; we have registered at the International Prospective Register of Systematic Reviews (PROSPERO, ID: CRD42022384127). The PRISMA checklist can be found in Supplementary Material File S1.

### 2.1. Search Strategy

Two reviewers (DJ and LZY) independently performed a thorough literature search on PubMed, Embase, Cochrane Library, and Scopus databases. The literature published since the inception of the database, until October 2022 was retrieved. The details of the PubMed search strategy are as follows: (“Liver Neoplasms” [Mesh] OR Neoplasms, Liver OR Liver Cancer OR Hepatic Neoplasm OR Hepatic Cancer OR Hepatocellular Cancer OR Hepatocellular Neoplasm OR Cancer of Liver) AND (“Thrombosis” [Mesh] OR Thrombus OR Blood Clot OR Clot, Blood OR portal vein tumour thrombus OR portal vein thrombosis OR PVTT OR Thrombus OR macroscopic vascular invasion) AND (“Chemoembolization, Therapeutic” [Mesh] OR Therapeutic Chemoembolization OR transcatheter arterial chemoembolization OR transarterial chemoembolization OR TACE OR Chemotherapy) AND (“c-Mer Tyrosine Kinase” [Mesh] OR tyrosine kinase inhibitor OR Sorafenib OR TKIS OR Lenvatinib OR regorafenib OR cabozantinib OR donafenib OR Apatinib OR Kinase, c-Mer Tyrosine OR Tyrosine Kinase, c-Mer OR c Mer Tyrosine Kinase OR c-Mer Proto-Oncogene Tyrosine Kinase OR c Mer Proto Oncogene Tyrosine Kinase OR MERTK). The search strategies for other databases can be found in Supplementary Material File S2.

### 2.2. Inclusion and Exclusion Criteria

The inclusion criteria were as follows: (1) studies that had patients with HCC and PVTT; (2) studies comparing TACE+TKI and TACE alone or TACE+another TKI; (3) tumor response, time to progression (TTP), overall survival (OS), and adverse events (AEs) were reported or calculated using related data; and (4) published in English language.

The exclusion criteria were as follows: (1) non-clinical studies, such as case reports, reviews, meta-analyses, letters, guidelines, conference papers, basic trials, and comments; (2) studies with data unrelated to TACE combined with TKIs or those that could not be extracted; and (3) articles lacking relevant outcome measures.

### 2.3. Data Extraction

After relevant articles were identified from the above databases, two reviewers (DJ and LZY) extracted and reviewed the data separately. Standardized tables were used to extract data from eligible articles. When required, a third reviewer (LR) was invited to settle the disagreements. The necessary data extracted from each study included: (1) primary author’s name, year of publication, journal of publication, and country of study; (2) study design, number of participants, sex, age, types of PVTT, tumor size, liver function (Child A/B/C), HCC stage (ECOG), virology, and alpha fetoprotein (AFP); (3) treatment strategy (including medication, dose, duration) and follow-up time; (4) overall survival (OS), time to progression (TTP), tumor response, objective response rate (ORR), disease control rate (DCR), and adverse events (AEs).

The primary end point was overall survival (OS), defined as the time from randomization or treatment to the death or the last follow-up. Secondary end points included TTP, ORR, and DCR. TTP was defined as the time interval from randomization or treatment to radiologic tumor progression. Tumor response was assessed according to the modified Response Evaluation Criteria in Solid Tumors (mRECIST), including complete response (CR), partial response (PR), progressive disease (PD), and stable disease (SD) [28]. The overall response rate (ORR) and disease control rate (DCR) were defined as CR+PR and CR+PR+PD, respectively. AEs were evaluated using National Cancer Institute Common Terminology Criteria for Adverse Events (CTCAE).

### 2.4. Quality Assessment

The quality of eligible literature was independently assessed by two authors (DJ and LZY). The modified Jadad scale was used for randomized controlled trials (RCTs) to indicate low (scores 1–3) and high (scores 4–7) quality [29]. The Newcastle–Ottawa Scale (NOS) was used to evaluate the quality of cohort studies, where scores of 1 to 4 indicated low quality and scores of 5 to 9 indicated high quality [30].

### 2.5. Statistical Analysis

Survival results, such as OS and TTP, were reported as hazard ratios (HRs) and 95% confidence intervals (CIs). ORR and DCR were calculated using the risk ratio (RR) and 95% CI. Qualitative heterogeneity was evaluated using I^2^ analysis. We adapted a fixed effects model when the I^2^ value was less than 50%; otherwise, it was random. Sensitivity analyses were performed by removing each study individually when heterogeneity was observed. For all outcomes, *p*-value < 0.05 meant statistical significance. All statistical analyses were conducted using RevMan5.4.

## 3. Results

### 3.1. Selection of Studies

**Figure 1:**
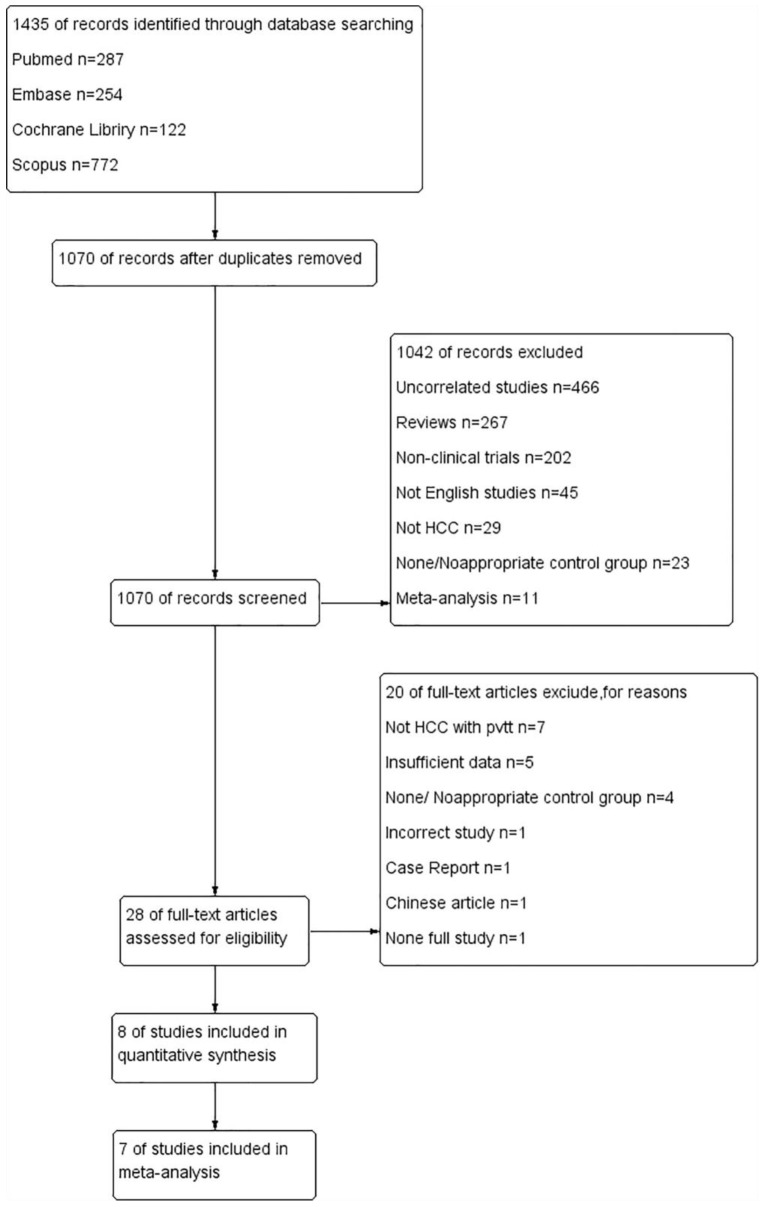
Flowchart for selection of included studies.

Following the search of multiple databases, a total of 1435 pieces of literature were identified for the initial screening. After automatic and manual checking, 365 duplicate studies were excluded. The titles and abstracts of the remaining articles were independently read by two investigators, and 28 potential articles were selected to carefully examine the full text. Finally, eight comparative, controlled articles were included in the systematic review [31,32,33,34,35,36,37,38], of which seven studies were included in the meta-analysis [34]. In a study by Sun et al., the baseline-related data were shown as HR (95%CI) of OS and TTP and the baseline characteristics did not show significant differences; the study was selected for meta-analysis (Figure 1). 

### 3.2. Study Characteristics and Quality Assessment

The articles published between 2014 and 2022 included one RCT and seven retrospective cohort studies. Sorafenib was used in three studies [34,35,36] that compared combination therapy with monotherapy, while apatinib was used in another three studies [31,32,33]. The remaining studies compared TACE plus lenvatinib with TACE plus sorafenib [37,38]. There were 2103 patients enrolled in this study, of whom 586 received combination therapy and 15,177 monotherapy. Most patients were in their fifties, and were infected with hepatitis B virus. Moreover, in the majority of patients, the baseline liver function was Child–Pugh A, indicating that liver function was well compensated. No significant difference in liver function was observed between the experimental and control groups. The remaining relevant baseline data are shown in Table 1.

Sorafenib (400 mg) was orally administered twice a day, while apatinib (500 mg) was administered once a day. However, the dose of lenvatinib was 8 mg or calculated based on the weight of the patients and administered orally, once a day. TKIs were administered for 3–11.4 months. Other TACE-related chemotherapy data are presented in Supplementary Material Table S1. 

RCTs were assessed using the modified Jadad scale, and cohort studies were assessed using the NOS scale. The results suggested that all studies included were of high quality. 

### 3.3. Efficacy of TACE Combined with Different TKI Agents

#### 3.3.1. Efficacy of Combination Therapy vs. Monotherapy

**Figure 2:**
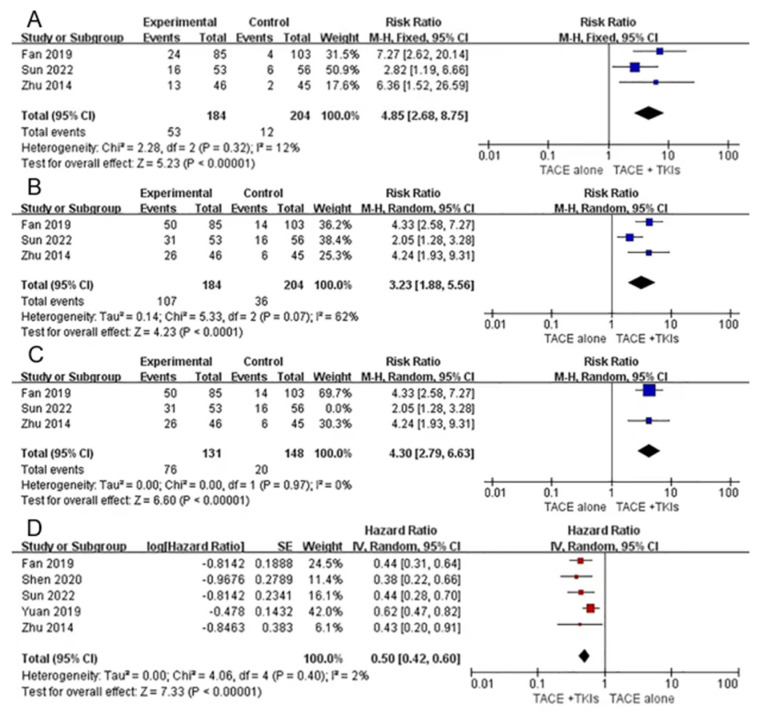
Forest plots of tumor response and overall survival for combination therapy vs. monotherapy. Outcomes: (**A**): objective response rate (ORR); (**B**): disease control rate (DCR); (**C**): sensitivity analysis; (**D**): overall survival (OS). TACE: transarterial chemoembolization; TKIs: tyrosine kinase inhibitors [31,33,36].

As described in Supplementary Material Table S2, three studies comparing combination therapy with monotherapy reported relevant data regarding ORR and DCR. Our meta-analysis demonstrated that compared with TACE alone, combination therapy with TACE and TKIs could significantly increase the ORR (RR = 4.85, 95% CI 2.68–8.75, Z = 5.23, *p* < 0.00001) with low heterogeneity (*p* = 0.23, I^2^ = 12%) and DCR (RR = 3.23, 95% CI 1.88–5.56, Z = 4.23, *p* < 0.0001) with high heterogeneity (*p* = 0.07, I^2^ = 62%). This indicated that combination therapy tended to have a better response in patients with HCC and PVTT. Sensitivity analysis by excluding each study individually suggested that Sun et al.’s study was the source of heterogeneity. After excluding this study, the final result favored combination therapy (RR: 4.30, 95%CI 2.79–6.63, *p* < 0.00001, I^2^ = 0%) (Figure 2).

All the studies reported relevant data on OS. The study by Wang et al. did not obtain HR (95% CI) for OS; therefore, this study was excluded from the meta-analysis. Meta-analysis demonstrated that combination therapy was superior to monotherapy in the treatment of HCC with PVTT (HR = 0.50, 95%CI 0.42–0.60, Z = 7.33, *p* < 0.00001). The heterogeneity was low (*p* = 0.40, I^2^ = 2%) (Figure 2).

Only three studies described relevant TTP data. The results suggest that combination therapy could delay tumor progression compared to TACE alone. 

#### 3.3.2. Efficacy of TACE+Lenvatinib vs. TACE+Sorafinib

A total of two articles [37,38] compared the efficacy of TACE plus lenvatinib with TACE plus sorafenib, and are described in Supplementary Material Table S2. Yang et al. reported relevant data regarding tumor response, which showed that the lenvatinib combination group was superior to the sorafenib combination group in ORR (60.7% vs.38.9%) but similar in DCR (96.4% vs. 96.3%).

**Figure 3:**
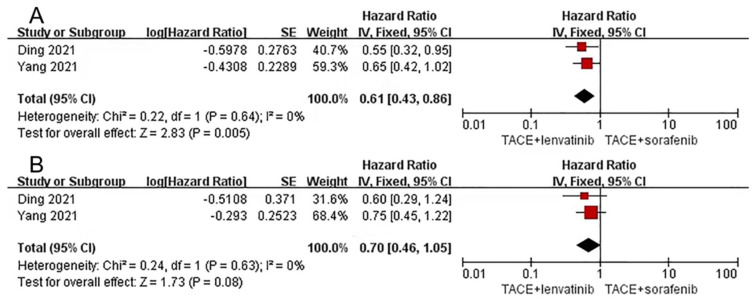
Forest plots for lenvatinib combination therapy vs. sorafenib combination therapy. Outcomes: (**A**): time to progression (TTP); (**B**): overall survival (OS). TACE: transarterial chemoembolization [37,38].

Our meta-analysis suggested that lenvatinib combination therapy delayed progression time for longer than sorafenib combination therapy (HR = 0.61, 95%CI 0.43–0.86, Z = 2.83, *p* = 0.005), and heterogeneity was low (*p*=0.64, I^2^=0%). However, no significant difference was found in OS (HR = 0. 70, 95% CI 0.46–1.05, Z = 1.73, *p* = 0.08) with low heterogeneity (*p* = 0.63, I^2^ = 0%) (Figure 3).

#### 3.3.3. Adverse Reactions

A total of six studies reported relevant data on toxicity of the combination treatment, which is shown in Supplementary Material Table S3. The most common AEs were hand–foot–skin reaction, diarrhea, hypertension, fatigue, and proteinuria. Adverse events were mostly TKI-related in the combination groups; however grade 3/4 AEs occurred with similar frequencies in different combinations. When AEs occurred, the dose of TKIs was generally reduced or even discontinued if necessary.

#### 3.3.4. Subgroup Analyses

A total of three of the included studies reported relevant data on various types of PVTT after combined treatment, and the results are shown in Supplementary Material Table S4. Among the included studies, Yang et al. analyzed outcomes according to the type of PVTT based on the Japanese standards (Vp1/Vp2: thrombus in minor and secondary branches; Vp3: primary portal vein branches; Vp4: portal trunk thrombus) [39], whereas other studies described the site of the cancer thrombus or defined PVTT using Cheng’s classification (Type I: tumor invasion of segmental branches of the portal vein or above; Type II: the right/left portal vein; Type III: the main portal vein trunk; Type IV: the superior mesenteric vein) [40]. After comparing the two classifications, Type I was similar to Vp1/Vp2, Type II was similar to Vp3, and Type III was analogous to Vp4. Cheng’s classification method was adopted.

**Figure 4:**
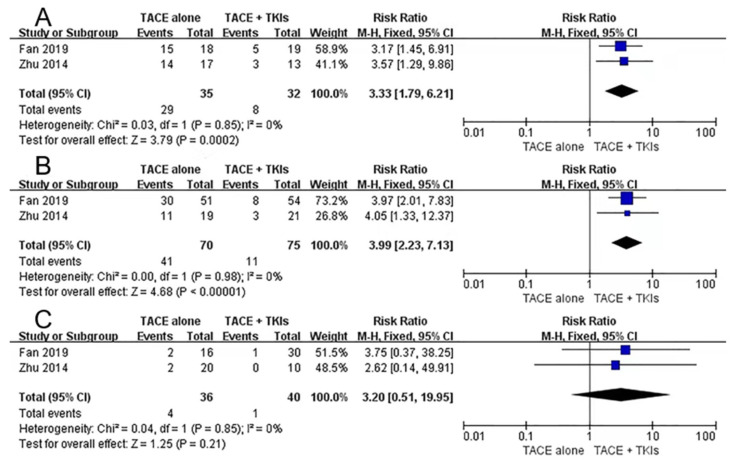
Subgroup analyses of disease control rate for combination therapy vs. monotherapy. (**A**): type I PVTT. (**B**): type II PVTT. (**C**): type III PVTT. TACE: transarterial chemoembolization; TKIs: tyrosine kinase inhibitors; PVTT: portal vein tumor thrombus [31,36].

Participants with type I and type II PVTT undergoing the combined therapy realized prolonged overall survival and time to disease progression. However, a similar benefit was not found in patients with type III PVTT. The meta-analysis suggested no statistically significant differences in type III patients with DCR (RR: 3.20, 95% CI 0.51–19.95, I^2^ = 0%, *p* = 0.21). Patients with types I (RR: 3.33, 95% CI 1.79–6.21, I^2^ = 0%, *p* = 0.0002) and II (RR: 3.99, 95% CI 2.23–7.13, I^2^ = 0%, *p* < 0.00001) had better disease control rates (Figure 4).

## 4. Discussion

Of those patients diagnosed with HCC for the first time, approximately 40% have PVTT [41]. Effective therapeutic measures are usually controversial and the prognosis is poor. Sorafenib and TACE combination therapy is not widely used due to its low response rate and drug resistance [42,43,44]. The efficacy of combination treatment with TACE and TKIs remains unclear.

The results of our meta-analysis provided evidence that combination therapy with sorafenib or apatinib improved ORR (RR = 4.85, 95% CI 2.68–8.75, Z = 5.23, *p* < 0.00001) and DCR (RR = 3.23, 95% CI 1.88–5.56, Z = 4.23, *p* < 0.0001), as well as prolonged the survival time of patients with advanced HCC and PVTT (HR = 0.50, 95%CI 0.42–0.60, Z = 7.33, *p* < 0.00001). Compared to sorafenib, combination therapy with lenvatinib seems to have no significant advantage in DCR (96.4% vs.96.3%) and OS (HR = 0.70 95% CI 0.46–1.05), although it performed better in ORR (60.7% vs. 38.9%) and TTP (HR = 0.61, 95% CI 0.43–0.86).

TACE can embolize tumor-feeding arteries and cause tumor necrosis. However, repeated TACE carries the risk of treatment failure and the impairment of liver function [45]. Tyrosine kinase inhibitors play an important role in tumor growth inhibition by acting on the VEGF pathway. Thus, the combined use of these two treatment modalities could provide a complementary effect. Our meta-analysis provides evidence supporting this hypothesis.

However, there are opposing views. A systematic review suggested that TACE plus sorafenib therapy provided no additive benefit for patients with HCC when compared with TACE plus placebo therapy [46]. A similar conclusion was reported in an RCT by Riccardo Lencioni [47]. There are more researchers who share our opinion. A retrospective analysis showed that the combination of TACE and sorafenib was better tolerated and improved the patient prognosis [48]. A network meta-analysis by Luo et al. demonstrated that sorafenib plus TACE was the most effective option after comparing TACE, sorafenib, hepatectomy, and any combination of the two [49]. Furthermore, lenvatinib, a multi-target TKI, acts on the VEGF receptor family. LAUNCH, an open-label, randomized, multicenter, parallel-group phase 3 trial, showed that combination therapy (TACE+lenvatinib group, *n* = 170) improved the ORR of patients (54.1% vs. 25.0%, *p* < 0.001) and prolonged OS (middle OS: 17.8 mouths vs. 11.5 mouths) when compared with lenvatinib monotherapy (*n* = 168) [50]. Apatinib, a TKI specifically targeting VEGFR-2, has shown definite efficacy in advanced liver cancer with PVTT in the real world [51]. A propensity score matching study showed that the TACE+apatinib group benefited more than the only TACE group and inhibited metastasis after TACE. Therefore, in combination with our study, we conclude that TACE combined with TKIs is an effective therapeutic measure for patients with advanced HCC and PVTT.

The relative efficacy of TACE with various TKIs remains unclear. Our study reported no significant difference between TACE+lenvatinib and TACE+sorafenib, based on limited evidence. More related studies have been reported in recent years. A 45-year-old patient with HCC received a combination therapy of TACE and sorafenib after hepatectomy, and had tumor progression due to sorafenib resistance and expression of VEGF. Fortunately, after adjustment to apatinib, the patient achieved disease control and eventually survived for 19 months [52]. At the same time, regorafenib combined with TACE showed definite efficacy in patients in whom TACE plus sorafenib or lenvatinib had failed [53]. However, regorafenib combination therapy was not included in our study.

In this study, subgroup analysis was performed based on the PVTT classification. The results showed no significant benefit for patients with type III PVTT between combination therapy and monotherapy, which may be related to the degree of tumor invasion of the blood vessels. Similar findings were reported by Zhang et al. [54].

Previous systematic reviews mostly focused on studies of single tyrosine kinase inhibitors combined with TACE, and our meta-analysis provided the first comprehensive clinical evidence for the use of various TKI agents in combination with TACE. However, our meta-analysis has some limitations. First, owing to the limited number of included studies (less than 10), we did not perform a publication bias test because of statistical limitations. Second, although the randomized trials included in the study scored 7 and the eight cohort studies scored 7–9, retrospective studies might have led to selection bias. Third, different chemotherapeutic agents may have also influenced the reliability of the results. Fourth, the sample size in some of the included studies was small (less than 100), which also led to selection bias or detection bias. Fifth, all the included studies were conducted in China, and the findings may not be generalizable to the European population. HCC is one of the most common neoplasms in China, and Chinese doctors have extensive experience with it. The reliability of the studies included in this review was high and may provide some evidence for the management of advanced HCC with PVTT.

## 5. Conclusions

This study demonstrated that TACE combined with sorafenib or apatinib is superior to TACE alone for patients with HCC and PVTT, and TACE combined with lenvatinib did not show additional therapeutic benefits; however TACE and lenvatinib combined, produced better ORR and TTP. Moreover, combination therapy has several advantages for type I and type II PVTT. It is necessary to design more multicenter, prospective randomized controlled trials with large sample sizes to clarify more effective treatment combinations.

## Figures and Tables

**Table 1:** Baseline characteristics of the studies.

Study	Journal	Country	Trail	Treatment	Patients	PVTT (I/II/III)	AGE (Year)	Sex (M/F)	Tumor Size (cm) (<5/>5)	Child–Pugh (A/B/C)	ECOG Ps (0/1–2)	Virology (HBV/HC V/Other)	AFP (mg/L) (≤400/ >400)	Score
Fan et al., 2019 [31]	Clinical Therapeutics	China	RT	T+A	85	18/51/16	49 (17–71)	68/17	27/58 (<7/≥7)	73/12/0	67/18	68/0/15	38/47	9
				T	103	19/54/30	50 (19–80)	71/32	23/80 (<7/≥7)	87/16/0	90/13	78/0/25	38/65	
Shen et al. 2020 [32]	Journal *	China	RT	T+A	40	27/13 (I–II/III–IV)	17/23 (<50/≥50)	38/2	9/31	33/7/0	NA	36/0/4	18/22	7
				T	280	208/72 (I–II/III–IV)	134/146 (≤50/>50)	265/15	28/252	228/52/0	NA	264/0/16	127/153	
Sun et al., 2022 ^#^ [33]	Journal of Oncology	China	RT	T+A	53	NA	NA	NA	NA	NA	NA	NA	NA	9
				T	56	NA	NA	NA	NA	NA	NA	NA	NA	
Wang et al., 2016 [34]	Medicine	China	RT	T+S	113	31/45/37	58/55 (≤50/>50)	77/36	29/84	110/3/0	NA	29/0/0	NA	7
				T	604	47/288/269	285/319 (≤50/>50)	534/70	79/525	567/37/0	NA	125/0/0	NA	
Yuanet al., 2019 [35]	BioMed Research International	China	RT	T+S	69	43/26 (I–II/III–IV)	51 (21–79)	59/10	8.39 ± 4.45	NA	NA	NA	123 ± 202	7
				T	429	182/247 (I–II/III–IV)	51 (18–84)	380/49	9.65 ± 3.25	NA	NA	NA	197 ± 251	
Zhu et al. 2014 [36]	Radiology	China	RT	T+S	46	17/19/10	48.4 ± 8.1	39/7	NA	39/7/0	22/24	38/5/3	NA	8
				T	45	13/21/11	51.9 ± 12.2	38/7	NA	39/6	20/25	40/1/4	NA	
Ding et al., 2021 [37]	Cancer	China	RCT	T+L	32	21/11	57 ± 11	25/7	25/7	22/10	NA	30/1/1	16/16	7
				T+S	32	25/7	56 ± 11	22/10	23/9	28/4	NA	29/3/0	14/18	
Yang et al., 2021 [38]	Frontiers in oncology	China	RT	T+L	59	34/16/9	54.05 ± 11.35	54/5	19/40 (<7/>7)	56/3/0	7/42	54/1/4	26/33	8
				T+S	57	29/17/11	56.18 ± 12.16	50/7	20/37 (<7/≥7)	55/2/0	6/51	49/3/5	23/34	

RT: retrospective study; RCT: randomized controlled trial; T: transarterial chemoembolization (TACE); S: sorafenib; A: apatinib; L: lenvatinib; PVTT: portal vein tumor thrombus; M: male; F: female; ECOG PS: Eastern Cooperative Oncology Group Performance Status; AFP: alpha fetoprotein; NA: not applicable. * Shen et al. [32] Journal of Cancer Research and Therapeutics. ^#^ The data of the study by Sun et al. [33] shown as the hazard ratio (95%CI) of overall survival and time to progression.

## Supplementary Materials

The following supporting information can be downloaded at: https://www.mdpi.com/article/10.3390/curroncol30010096/s1, Supplementary Material File S1: The PRISMA checklist [27]; Supplementary Material File S2: The search strategies for Embase, Scopus, Cochrane Library databases; Supplementary Material Table S1: The characteristics of the combined therapy; Supplementary Material Table S2: Efficacy of hepatocellular carcinoma with portal vein tumor thrombus; Supplementary Material Table S3: Adverse events; Supplementary Material Table S4: The outcomes of patients treated with transarterial chemoembolization and tyrosine kinase inhibitors combination therapy for various types of portal vein tumor thrombus.
