# Supplementary material for: Efficacy of Transarterial Chemoembolization Combined with Tyrosine Kinase Inhibitors for Hepatocellular Carcinoma Patients with Portal Vein Tumor Thrombus: A Systematic Review and Meta-Analysis
Source: Curr Oncol. 2023 Jan 16;30(1):1243–54. doi: 10.3390/curroncol30010096 (PMC9858211; doi:10.3390/curroncol30010096)
Supplement: Supplementary file 1 [file curroncol-30-00096-s001.zip › Supplementary Material File S2.pdf]

Search Strategies

EMBASE:

#1 'liver tumor'/exp

#2 'hepatic tumor':ti,ab,kw OR 'hepatic tumour':ti,ab,kw OR 'hepatocellular neoplasia':ti,ab,kw OR 'hepatocellular neoplasm':ti,ab,kw OR 'hepatocellular tumor':ti,ab,kw OR 'hepatocellular tumorigenesis':ti,ab,kw OR 'hepatocellular tumour':ti,ab,kw OR 'hepatocyte tumorigenesis':ti,ab,kw OR 'liver cell tumor':ti,ab,kw OR 'liver cell tumour':ti,ab,kw OR 'liver neoplasia':ti,ab,kw OR 'liver neoplasm':ti,ab,kw OR 'liver neoplasma':ti,ab,kw OR 'liver neoplasms':ti,ab,kw OR 'liver tumorigenesis':ti,ab,kw OR 'liver tumour':ti,ab,kw OR 'neoplasia of the liver':ti,ab,kw OR 'neoplasm of the liver':ti,ab,kw OR 'neoplastic hepatocellular':ti,ab,kw OR 'neoplastic liver':ti,ab,kw OR 'tumor of the liver':ti,ab,kw OR 'tumor liver':ti,ab,kw OR 'tumour, liver':ti,ab,kw

#3 #1 OR #2

#4 'tumor thrombus'/exp

#5 'cancer thrombus':ti,ab,kw OR 'cancer-associated thrombosis':ti,ab,kw OR 'cancerous thrombosis':ti,ab,kw OR 'cancerous thrombus':ti,ab,kw OR 'carcinothrombosis':ti,ab,kw OR 'neoplastic thrombosis':ti,ab,kw OR 'neoplastic thrombus':ti,ab,kw OR 'thrombosis,tumor':ti,ab,kw OR 'thrombosis, tumour':ti,ab,kw OR 'thrombus, cancer':ti,ab,kw OR 'tumor thrombosis':ti,ab,kw OR 'tumoral thrombosis':ti,ab,kw OR 'tumoral thrombus':ti,ab,kw OR 'tumorthrombosis':ti,ab,kw OR 'tumorthrombus':ti,ab,kw OR 'tumothrombus':ti,ab,kw OR 'tumour thrombosis':ti,ab,kw OR 'tumour thrombus':ti,ab,kw OR 'tumoural thrombosis':ti,ab,kw OR 'tumoural thrombus':ti,ab,kw

#6 #4 OR #5

#7 'chemoembolization'/exp

#8 'arterial chemoembolisation':ti,ab,kw OR 'arterial chemoembolization':ti,ab,kw OR 'chemoembolisation':ti,ab,kw OR 'chemoembolization, therapeutic':ti,ab,kw OR 'therapeutic chemoembolization':ti,ab,kw OR 'transarterial chemoembolisation':ti,ab,kw OR 'transarterial chemoembolization':ti,ab,kw OR 'transcatheter arterial chemoembolisation':ti,ab,kw OR 'transcatheter arterial chemoembolization':ti,ab,kw OR 'transcatheter chemoembolisation':ti,ab,kw OR 'transcatheter chemoembolization':ti,ab,kw OR 'transcatheter oily chemoembolisation':ti,ab,kw OR 'transcatheter oily chemoembolization':ti,ab,kw OR 'tace':ti,ab,kw

#9 #7 OR #8

#10 'tyrosine kinase inhibitor':ti,ab,kw OR 'sorafenib':ti,ab,kw OR 'tkis':ti,ab,kw OR 'lenvatinib':ti,ab,kw OR 'regorafenib':ti,ab,kw OR 'cabozantinib':ti,ab,kw OR 'donafenib':ti,ab,kw OR 'apatinib':ti,ab,kw

#11 #3 AND #6 AND #9 AND #10

SCOPUS

((TITLE-ABS-KEY (liver AND neoplasms) OR TITLE-ABS-KEY (cancer AND of AND liver) OR TITLE-ABS-KEY (hepatocellular AND cancer) OR TITLE-ABS-KEY (hepatic AND cancer) OR TITLE-ABS-KEY (hepatic AND neoplasm) OR TITLE-ABS-KEY (liver AND cancer) OR TITLE-ABS-KEY (neoplasms, AND liver) OR TITLE-ABS-KEY (cancer AND of AND liver) OR TITLE-ABS-KEY (hepatocellular AND neoplasm))) AND ((TITLE-ABS-KEY (thrombosis) OR TITLE-ABS-KEY (portal AND vein AND tumour AND thrombus) OR TITLE-ABS-KEY (blood AND clot) OR TITLE-ABS-KEY (clot, AND blood) OR TITLE-ABS-KEY (portal AND vein AND thrombosis) OR TITLE-ABS-KEY (pvtt) OR TITLE-ABS-KEY (macroscopic AND vascular AND invasion) OR TITLE-ABS-KEY (thrombus))) AND ((TITLE-ABS-KEY (chemoembolization, AND therapeutic) OR TITLE-ABS-KEY (therapeutic AND chemoembolization) OR TITLE-ABS-KEY (transcatheter AND arterial AND chemoembolization) OR TITLE-ABS-KEY (transarterial AND chemoembolization) OR TITLE-ABS-KEY (tace) OR TITLE-ABS-KEY (chemotherapy))) AND ((TITLE-ABS-KEY (tyrosine AND kinase AND inhibitor) OR TITLE-ABS-KEY (sorafenib) OR TITLE-ABS-KEY (tkis) OR TITLE-ABS-KEY (lenvatinib) OR TITLE-ABS-KEY (regorafenib) OR TITLE-ABS-KEY (cabozantinib) OR TITLE-ABS-KEY (apatinib) OR TITLE-ABS-KEY (donafenib)))

Cochrane library

#1 MeSH descriptor: [Liver Neoplasms] explode all trees

#2 Hepatic Cancer OR Hepatic tumor OR Hepatic Neoplasm OR Hepatocellular Cancer OR Hepatocellular tumor OR Hepatocellular Neoplasm OR Liver Cancer OR Liver tumor OR Cancer, Liver OR Cancers, Hepatocellular OR Cancer, Hepatic OR Cancer of the Liver OR Cancer, Hepatocellular OR Cancers, Hepatic OR Cancers, Liver OR Cancer of Liver OR Neoplasm, Hepatic OR Neoplasm, Liver OR Neoplasms, Liver OR Cancer, Hepatic OR Cancer of the Liver Liver Neoplasm OR Neoplasms, Hepatic OR hepatic tumour OR hepatocellular tumorigenesis OR hepatocyte tumorigenesis OR liver cell tumor OR liver neoplasm OR liver tumour OR neoplastic Hepatocellular OR neoplastic liver OR tumor of the liver OR neoplastic liver OR hepatocellular tumorigenesis OR hepatocyte tumorigenesis OR liver cell tumour OR liver neoplasia OR liver neoplasma OR liver tumorigenesis OR neoplasia of the liver

#3 #1 OR #2

#4 MeSH descriptor: [Thrombosis] explode all trees

#5 thrombus OR Blood Clot OR Clot, Blood OR portal vein tumour thrombus OR portal vein thrombosis OR PVTT OR macroscopic vascular invasion OR thrombosis OR carcinothrombosis OR tumorthrombosis OR tumorthrombus

#6 #4 OR #5

#7 Chemoembolization OR chemoembolisation OR arterial chemoembolization Therapeutic OR therapeutic chemoembolization OR transcatheter arterial chemoembolization OR transarterial chemoembolization OR TACE OR Chemotherapy OR transarterial chemoembolisation OR transcatheter chemoembolization OR transcatheter oily chemoembolization OR arterial chemoembolisation OR chemoembolization, therapeutic OR transarterial chemoembolisation OR transcatheter arterial chemoembolisation OR transcatheter chemoembolisation OR transcatheter oily chemoembolisation

#8 tyrosine kinase inhibitor OR sorafenib OR tki OR lenvatinib OR regorafenib OR cabozantinib OR donafenib OR apatinib

#9 #3 AND #6 AND #7 AND #8
