# Supplementary material for: Efficacy of Transarterial Chemoembolization Combined with Tyrosine Kinase Inhibitors for Hepatocellular Carcinoma Patients with Portal Vein Tumor Thrombus: A Systematic Review and Meta-Analysis
Source: Curr Oncol. 2023 Jan 16;30(1):1243–54. doi: 10.3390/curroncol30010096 (PMC9858211; doi:10.3390/curroncol30010096)
Supplement: Supplementary file 1 [file curroncol-30-00096-s001.zip › Supplementary Material Tables S1-S4.pdf]

Supplementary Material Table S1:The characteristics of the combined therapy

Study	TKI agents	NO. TKI (months)	Chemotherapeutic agents	NO.TACE
Fan et al.2019 [31]	Apatinib:500 mg qd	NA	300 mg carboplatin	NA
Shen et al. 2020 [32]	Apatinib:500 mg qd	3.7	30–50 mg lobaplatin,30–50 pirarubicin	NA
Sun et al. 2022 [33]	Apatinib:500mg qd	11.4 (95%CI:9.8–13.)	10–40mg doxorubicin hydrochloride	NA
Wang et al. 2016 [34]	Sorafenib:400mg bid	NA	20–60mg doxorubicin hydrochloride, 5mg cisplatin	NA
Yuan et al. 2019 [35]	Sorafenib:400 mg bid	NA	1.0 g 5-fluorouracil 150 mg oxaplatin	Mean 3times (range1–11)
Zhu et al. 2014 [36]	Sorafenib:400mg bid	11.0 (95%CI:8.2–13.8)	20–60 mg Doxorubicin 20–50 mg lobaplatin	Mean3.6 times (range1–8)
Ding et al. 2021 [37]	Sorafenib:400 mg bid lenvatinib:≤60 kg/ Child B 8 mg qd:≥60kg,12 mg qd	T+L:6.9(range1.3–20) T+S:3.0(range1.1–12.6)	50 mg epirubicin	NA
Yang et al. 2021 [38]	Lenvatinib:8 mg qd Sorafenib：400 mg bid	NA	40–45 mg epirubicin	T+L:2.09 ±0.198 T+S:2.03 ±0.118

TKI: tyrosine kinase inhibitor; T: transarterial chemoembolization(TACE); S:sorafenib; L: lenvatinib; NA: not applicable; NO: number; qd: once a day; bid: twice a day.

Supplementary Material Table S2:Efficacy of hepatocellular carcinoma with portal vein tumor thrombus

Study	Treatment	Tumor response				ORR (%)	DCR (%)	6-month Survival rate (%)	1-year Survival rate (%)	Median OS (months) (95%CI)	Median TTP (months) (95%CI)
		CP	PR	SD	PD						
Fan et al. 2019 [31]	T+A	0	24	26	35	28.2	59	76.4	29.4	12(10.3–13.7)	6.1(4.9,7.3)
	T	0	4	10	89	3.9	14	60.2	7.8	7(6.4–7.6)	3.7(3.1–4.4)
Shen et al. 2020 [32]	T+A	NA	NA	NA	NA	NA	NA	85.7	63.6	18.2	NA
	T	NA	NA	NA	NA	NA	NA	60.9	40.6	7.1	NA
Sun et al. 2022 [33]	T+A	NA	NA	NA	NA	30.2	58.5	86.7	58.5	15	7
	T	NA	NA	NA	NA	10.7	28.6	57.5	14.2	7	3
Wang, et al.. 2016 [34]	T+S	NA	NA	NA	NA	NA	NA	67.9	37.36	8.92(7.86–10.97)	NA
	T	NA	NA	NA	NA	NA	NA	41.6	24.16	4.79(4.07–5.45)	NA
Yuan et al. 2019 [35]	T+S	NA	NA	NA	NA	NA	NA	86.9	46.7	13.0(9.2–16.8)	NA
	T	NA	NA	NA	NA	NA	NA	55.5	22.57	6.0(5.4–6.6)	NA
Zhu et al. 2014 [36]	T+S	0	13	13	20	28.3	57	82.6	45.7	11.0(7.8–14.2)	6.0(4.9–7.1)
	T	0	2	4	39	4.4	13	60.0	17.8	6.0 (4.9–7.1)	3.0(2.2–3.8)
Ding et al. 2021 [37]	T+L	NA	NA	NA	NA	NA	NA	93.5	54.1	14.5(8.4–20.6)	4.7(2.0–7.4)
	T+S	NA	NA	NA	NA	NA	NA	82.0	45.0	10.8 (7.7–13.9)	3.1(2.7–3.5)
Yang et al. 2021 [38]	T+L	6	28	20	2	60.7	96.4	81.4	40.7	16.4(10.9,21.8)	8.4(7.07–15.2)
	T+S	3	18	31	2	38.9	96.3	78.9	40.4	12.7(10.8,17.9)	7.43(5.63–9.03)

CR: complete response; PR: partial response; SD: stable disease; PD: progressive disease; ORR: CR+PR DCR; CDR: CR+PR+SD

OS: overall survival; TTP: time to progression; T:transarterial chemoembolization(TACE); S:sorafenib; A:apatinib; L: lenvatinib; NA: not applicable.

Supplementary Material Table S3:Adverse events

Adverse events	Fan et al.2019 [31]	Shen et al.2020 [32]	Sun et al. 2022 [33]	Wang et al. 2016 [34]	Yuan et al. 2019 [35]	Zhu et al. 2014 [36]	Ding et al. 2021 [37]		Yang et al. 2021 [38]		Total
							T+L	T+S	T+L	T+S	
HFSR (n/%)	45 (52.9)	27 (67.5)	44 (83)	NA	NA	37 (80)	4 (12.5)	8 (25)	10 (17.5)	16 (30.7)	191
Hemorrhage of digestive tract(n/%)	1 (1.2)	NA	4 (7.5）	NA	NA	4 (9)	NA	NA	5 (8.7)	3 (5.8)	17
Diarrhea(n/%)	1 (22.1)	9 (22)	10 (18.9)	NA	NA	33 (72)	13 (40.6）	10 (31.3)	10 (17.5)	13 (24.8)	99
Hypertension(n/%)	43 (50.6)	13 (32.5)	24 (45.3)	NA	NA	6 (13)	19 (59.4）	11 (34.4)	8 (14.0)	4 (7.7)	128
Fatigue(n/%)	8 (9.4)	5 (12.5)	6 (11.3)	NA	NA	13 (28)	18 (56.3）	14 (43.8)	NA	NA	64
Liver dysfunction (n/%)	NA	NA	NA	NA	NA	NA	28(87.5）	29 (90.6)	7 (12.2)	4 (7.7)	49
Rash/Desquamation (n/%)	NA	NA	NA	NA	NA	NA	4 (12.5)	11 (34.4)	3(5.2)	4 (7.7)	22
Oral ulcer(n/%)	14 (16.5)	5 (12.5)	3 (5.7)	NA	NA	NA	NA	NA	4 (7.0)	1 (1.9)	27
Voice change(n/%)	10 (11.8)	5 (12.5)	3 (5.7)	NA	NA	1(2)	8 (25）	1 (3.2)	1 (1.7)	1 (1.9)	30
Proteinuria(n/%)	26 (30.6)	17 (42.5)	10 (18.9)	NA	NA	NA	10 (31.3）	4 (6.3)	NA	NA	67
Headache (n/%)	18 (21.2)	NA	NA	NA	NA	NA	NA	NA	NA	NA	18
cough/fever(n/%)	16 (18.8)	NA	NA	NA	NA	NA	23 (71.9）	16 (50.0）	NA	NA	55
Nausea(n/%)	NA	NA	NA	NA	NA	NA	25 (78.1)	18 (56.5)	8 (14.0)	4 (7.7)	55
Anemia (n/%)	8 (9.4)	NA	NA	NA	NA	NA	12 (37.5)	3 (9.6)	NA	1 (1.9)	24

Abdominal pain(n/%)	21 (24.7)	NA	NA	NA	NA	NA	23 (71.9）	14 (43.6)	NA	NA	58
Adverse events (grade3or4/any grade)	39/238	19/76	7/104	NA	NA	16/110	38/350	35/256	13/57	10/52	176/1243

HFSR: hand–foot skin reaction. T:transarterial chemoembolization(TACE); S:sorafenib; L: lenvatinib; NA: not applicable.

Supplementary Material Table S4:The outcomes of patients treated with transarterial chemoembolization and tyrosine kinase inhibitors combination therapy for various types of portal vein tumor thrombus.

Study	Treatment	Type of PVTT	Patients	Median TTP (months)	Median OS (months)	DCR (%)
Fan et al. 2019 [31]	T+A	Type I	18	8.3	13.7	83
		Type II	51	6.9	12.2	59
		Type III	16	1.2	5.4	13
	T	Type I	19	4.6	7.2	26
		Type II	54	4.2	7.5	15
		Type III	30	2.3	4.5	3
Wang et al. 2016 [34]	T+S	Type I	31	NA	12.010	NA
		Type II	45	NA	8.920	NA
		Type III	37	NA	6.960	NA
	T	Type I	47	NA	9.280	NA
		Type II	288	NA	4.900	NA
		Type III	269	NA	3.980	NA
Zhu et al. 2014 [36]	T+S	Type I	17	7	15	82
		Type II	19	6	13	58
		Type III	20	0	3	10
	T	Type I	13	5	10	23
		Type II	21	3	6	14

		Type III	10	0	3	0

PVTT：portal vein tumor thrombus; TTP: time to progression; OS: overall survival; DCR: disease control rate; T:transarterial chemoembolization(TACE); A:apatinib;S:sorafenib; NA: not applicable.
